# Saccade length consistency during reading and shape-scanning

**DOI:** 10.3758/s13414-026-03289-6

**Published:** 2026-06-03

**Authors:** Thomas Fabian

**Affiliations:** Department of History and Social Sciences, Technical University of Darmstadt, Residenzschloss 1, 64283 Darmstadt, Germany

**Keywords:** Eye movements and visual attention, Reading, Visual search

## Abstract

Human gaze behaviour provides insights into the mental processes underlying the execution of a task. As reading involves visual sampling and language processing, various studies investigate how the linguistic information of texts influences visual behaviour. However, established measures of human visual behaviour are dependent on the exact configuration of the text stimuli and the nonlinguistic stimuli used for comparison, leaving systematic stimulus-independent differences largely unknown. Here, we show that relative saccade length distributions reveal similarities and differences in gaze dynamics during reading and shape-scanning. In a within-subject design, participants read texts and scanned a spatially matched array of geometric shapes. We find that the lengths of consecutive saccades in target direction are more consistent during reading than during shape-scanning, suggesting that saccadic planning is more constrained during reading. The consistency of eye movements opposite to the target direction does not differ for the experimental conditions. These results consolidate findings from neurocognitive studies on the vision–language interface and suggest how underlying neural structures manifest themselves in observable visual behaviour. Furthermore, the results indicate that relative saccade lengths could present a new measure for investigating how linguistic processing influences human visual behaviour during reading. More broadly, the results suggest that analysing gaze behaviour dynamics via relative saccade lengths might provide novel insights into similarities and differences across tasks.

## Introduction

Over the past decades, eye-tracking research has been utilised to investigate human behaviour and cognition. Research in this area focuses on various topics, including viewing images and reading text. Eye-tracking helps in understanding online shopping behaviour (Hwang & Lee, 2018), explaining differences in car-driving behaviour (Underwood et al., 2003), providing insights into infants’ cognitive development (Constantino et al., 2017; Maurer, 1983), and even diagnosing Alzheimer’s disease (Readman et al., 2021). Eye-tracking is a universal tool, as humans must constantly move their eyes to gather visual information. Since acuity is highest in the centre of the visual field, one has to continually reposition the eyes to see objects sharply and to be able to take in information. While the eyes are relatively still, called a fixation, we take in information (Rayner, 2009a). During a fixation, it is also necessary to decide which point to fixate on next (Kümmerer & Bethge, 2021; McConkie et al., 1988). This decision is based on the judgment at which point one can gather as much new information as possible with the smallest possible eye movement.

A broad area of application for eye-tracking is explaining human reading behaviour. Understanding how people read texts is of particular interest since it involves different levels of cognition. Humans must perceive the letters and words (Rayner, 1998), comprehend their meaning (Perfetti, 2007), and integrate the information into a mental model (Bower & Morrow, 1990; Garnham & Oakhill, 1996). This combination of involved processes renders reading a complex and challenging subject for research. Much progress in reading research has been made, among others, by Keith Rayner and his colleagues (Clifton et al., 2016; Rayner, 1975a, b, 1998, 2009a; Rayner & McConkie, 1976). They have found that when reading, one can perceive information from peripheral vision (Rayner, 1975a, 1998, 2009a). This information, the meaning of individual words or just the cue of a word's length, helps to perform the following eye movement as efficiently as possible. They also found that the range of this ability is spatially limited, called the perceptual span (McConkie & Rayner, 1975, 1976; Rayner, 1975b). The size of the perceptual span depends on various factors, such as reading experience or age (Rayner, 1986; Rayner et al., 2009). It is also claimed that the perceptual span is not dictated by limitations of visual acuity (Miellet et al., 2009; Rayner et al., 2010).

In reading research, the information of the text and individual words is usually claimed as the central argument, if not the only argument, for the decision to make an eye movement. For example, the influential E-Z Reader model (Pollatsek et al., 2006; Rayner, 1998; Rayner et al., 2004; Reichle et al., 1998, 2003) is based on the assumption that the movement of the eyes when reading a text is prompted solely by the processing of the linguistic information of the text. According to reading research, texts are unique due to the linguistic information that fundamentally distinguishes reading from other tasks. Experimental findings suggest no correlations between human visual behaviour during tasks such as reading and visual search (Andrews & Coppola, 1999; Rayner, 2009a; Rayner et al., 2007). Rayner (2009a) reports that saccades during silent reading in an alphabetic writing system have an average length of 2° of visual angle and fixation durations are around 225–250 ms. For visual search, saccade lengths are longer and fixation durations have more variance with 3° of visual angle and 180–275 ms, respectively. In scene perception, saccade and fixation durations are longer than during reading with 4–5° of visual angle and 260–330 ms. Rayner (2009a) also notes that these values are only averages and actual values have high variability. In the case of reading, saccade lengths can vary between 0.25° and 5° of visual angle and fixation durations can vary between 50–75 ms and 500–600 ms. For visual search tasks, the actual values are highly dependent on the task’s complexity and the stimulus layout.

Beyond reading research, however, there are also results from cognitive research that explain reading behaviour differently than by the linguistic information of the text. Pelli et al. (2007) note that the visual span can be explained by crowding, a phenomenon in which similar objects, such as letters, are ambiguated in the visual periphery (for a review on crowding, see Rosenholtz, 2016). The occurrence of crowding depends on the eccentricity of the objects and their distance from each other (Bouma, 1970). According to Pelli et al. (2007), the visual span is the uncrowded part of the peripheral view, which is similar to the perceptual span in reading research. According to the findings of Pelli et al. (2007), the visual span is independent of linguistic information. Similarly, research by Vitu and colleagues has shown that visuo-motor constraints dominate reading behaviour, and linguistic processing only modulates gaze behaviour (Albrengues et al., 2019; Vitu, 2011). Albrengues et al. (2019) show with an eye-tracking experiment on natural sentence reading that low-level visuo-motor factors like word length and launch-site distance are the strongest predictors of saccade landing positions, whereas linguistic top-down factors like word frequency and predictability only cause small modulations. Vitu (2011) summarises 40 years of research on eye movements during reading and argues that mainly low-level processes predict eye movements, since word length and fixation position are consistently the two most important factors, in contrast to the minor significance of high-level visual attention and selection processes.

In addition to cognitive research, neuroscience provides findings on how neural functioning can influence reading behaviour. Experimental findings suggest the existence of a basic rhythm of attention in the human brain (Bolger et al., 2013; Buzsáki, 2006; Fiebelkorn & Kastner, 2019; Lakatos et al., 2009). This rhythm optimises visual information perception by providing an efficient ratio of information sampling and attention shifting. Within the 3–8 Hz rhythm, information intake is inhibited, and the execution of an eye movement is facilitated. Since this occurs independently of the viewed object, eye movements appear to have an automatic component that is driven solely by oculomotor physiology. The influence of this automatic part on human gaze behaviour has been demonstrated in many tasks and stimulus types using relative eye-movement lengths (Fabian, 2025), indicating a universal part of gaze behaviour. However, it is not clear exactly how this oculomotor influence affects visual behaviour during reading and whether the linguistic information of the text modifies the distribution of relative eye-movement lengths in any way.

We apply the oculomotor view of cognitive science to reading. Since research on reading explains human reading behaviour differently from cognitive science and neuroscience, combining these views may offer a more holistic understanding of the vision–language interface of human vision. This work examines how gaze behaviour differs between reading and scanning a nonlinguistic display. To this end, we create stimuli with arrays of geometric shapes whose layout mimics the reading stimuli. In the experiment, participants scan the shapes as if they were reading a text to count the number of a specific shape–colour combination. In this within-subjects design, we calculate the standard measures of eye-movement behaviour, saccade lengths, and fixation durations, to assess how gaze behaviour differs between reading and shape-scanning. Mindless reading of *z*-strings suggests that scanning unreadable strings leads to longer fixation durations than normal reading and comparable average saccade lengths (Rayner & Fischer, 1996).

However, established measures, such as fixation durations and saccade lengths, strongly depend on the experiment design and exact stimulus configuration (Legge et al., 1985; Parkhurst et al., 2002; Slattery & Rayner, 2013). To minimise the effect of stimulus configuration on comparisons of reading to other tasks, we apply a new measure of human visual behaviour—relative saccade length distributions (Fabian, 2025). Dividing the length of an eye movement by the length of the previous eye movement yields a distribution that persists across various tasks, observers, and stimuli. Thus, this measure might provide a novel view on the vision–language interface of human visual behaviour that, unlike saccade lengths and fixation durations, is independent of the specific experiment design and stimulus configuration. We perform more fine-grained analyses of relative eye-movement lengths than Fabian (2025) by distinguishing eye movements in reading direction from those that serve to revisit words (i.e., saccades and regressions). Given that distributions of relative eye-movement lengths can still exhibit slight deviations despite their common power-law distributed tail (Fabian, 2025), the separation into saccades and regressions enables a more precise investigation of the vision–language interface (i.e., how reading behaviour differs from scanning a nonlinguistic display).

As an extension of Fabian's work (2025), we propose that relative eye-movement length distributions allow one to evaluate the consistency of eye-movement lengths. Assuming that, on average, eye movements do not get longer or shorter over longer periods of performing a task, the average relative length for the *n* relative eye-movement lengths ${{\ell}}_{1},\dots ,{{\ell}}_{n}$ should converge towards 1 for large *n*:

$$\sqrt[n]{\prod_{i=1}^{n}{{\ell}}_{i}}\approx 1.$$

Given this axiom of relative eye-movement lengths, which we test in our empirical data, we can use the peak position of the distribution to gain insights into the dynamics of gaze behaviour. A peak probability closer to 1 indicates that relative eye movements are more densely distributed around the value 1, and by that, successive movements are more similar in length. By subdividing the eye movements into saccades and regressions, we can understand more precisely how reading affects the dynamics of the two eye movement types compared with shape-scanning. Relative saccade lengths could present a novel measure for comparing spatial saccade-to-saccade dynamics in human visual behaviour, as they reflect the relations between saccade lengths and introduce sequential spatial dependencies. Calculating the variability of absolute saccade lengths does not achieve the same. Performing many small saccades followed by many large saccades would yield a high variability, but most successively performed saccades do not actually differ much in length. Relative saccade length distributions, however, will reveal consistent gaze dynamics, as saccade-to-saccade differences are small.

The primary research question addressed in this work concerns the vision–language interface of vision: How can one meaningfully compare human visual behaviour when reading texts and scanning nonlinguistic displays? To answer this question, we conduct an experiment with reading stimuli and spatially matched shape-scanning stimuli. Then, we first calculate standard measures of eye-movement behaviour and compare the results with previous findings from similar studies. Second, we determine relative saccade length distributions for the saccades and regressions performed during the tasks to assess the consistency of eye-movement lengths. We investigate if and how the eye movement consistency differs between the tasks without a priori hypotheses. To utilise the relative eye-movement length distributions for evaluating the consistency of eye-movement lengths, a secondary research question must be answered: Do average relative eye-movement lengths converge towards the value 1? To answer the secondary research question, the average relative lengths of saccades and regressions are calculated during both reading and shape-scanning. As we described before, we hypothesise that the average relative lengths converge towards 1 as it should be assumed that eye-movement lengths do not systematically increase or decrease over longer periods of performing a task. Only if the average relative eye-movement lengths converge to 1, we can interpret the distribution’s peak probability with regard to the consistency of gaze behaviour.

## Methods

### Stimuli

The reading stimuli serve as a baseline for everyday reading behaviour. We select the 26 texts for the reading stimuli based on several criteria. All text samples are from novels by gender-balanced authors. According to their publication date, the novels are equally distributed from 1900 to 2016. All text samples in the experiment have 96 to a maximum of 105 words, with a mean value of 100. We present the text stimuli with a line spacing of 2 and a font size of 18 in the monospaced font Courier New so that all characters convey the same graphemic information. Beyond these structural aspects, we also impose content-related conditions on the text samples. They must deal with a fact or a topic that a multiple-choice question can test. For this reason, we only select samples that focus on describing a person or an environment. Additionally, all text samples contain neither a first-person perspective nor literal speech and are self-contained (i.e., understanding them requires no background information from the rest of the novel). We present the texts in their original English version. Although the participants are German native speakers and eye movements during nonnative reading can differ from native reading (Berzak & Levy, 2023), we decide to present the English originals for two reasons. First, we avoid any alterations of the original texts during translation. Second, using English texts provides better comparability to other English reading studies by matching language-dependent factors like word-length distributions (Kuperman et al., 2024; Siegelman et al., 2022; Wichmann & Holman, 2023), which have been shown to differ particularly for English and German (Sigurd et al., 2004).

The creation of our shape-scanning stimuli is based on the text stimuli, similar to the Landolt paradigm (Corbic et al., 2007; Günther et al., 2012; Hillen et al., 2013; Zschornak et al., 2008, 2012), which is used to create text-like stimuli without lexical, syntactic, or semantic information. In the Landolt paradigm, nonlinguistic versions of text stimuli are created by replacing each character with a circle. Some of these circles, also called Landolt rings (Landolt, 1899), have an opening. Participants are instructed to scan the stimuli like they would read a text and press a button whenever they spot a Landolt ring with an opening. Previous studies have compared eye movements during reading and Landolt-like *z*-string scanning (Rayner & Fischer, 1996; Vitu et al., 1995), in which most letters in a text are replaced by the letter *z* and few by the letter *c*. The participants’ task for these stimuli is to respond each time they find the letter *c*. Whereas Vitu et al. (1995) report that oculomotor measures are similar for *z*-scanning and reading, Rayner and Fischer (1996) refine the experimental conditions to highlight that reading leads to shorter fixations than *z*-scanning and comparable average saccade lengths. Other studies employ the Landolt paradigm to show how spacing and frequency effects during reading can be replicated without linguistic information (Vanyukov et al., 2012; Wang et al., 2021), but they do not directly compare reading with the Landolt task. We also use the Landolt paradigm in our experiment because other pseudoreading approaches do not completely remove linguistic processing from the task. For example, when participants are instructed to search for a specific word or count the occurrences of a particular letter rather than reading the text, they still employ linguistic processing in parafoveal vision (Schotter et al., 2012). Similarly, presenting words with jumbled letters will still allow participants to process the letter strings as the original words (Rayner et al., 2006).

**Fig. 1:**
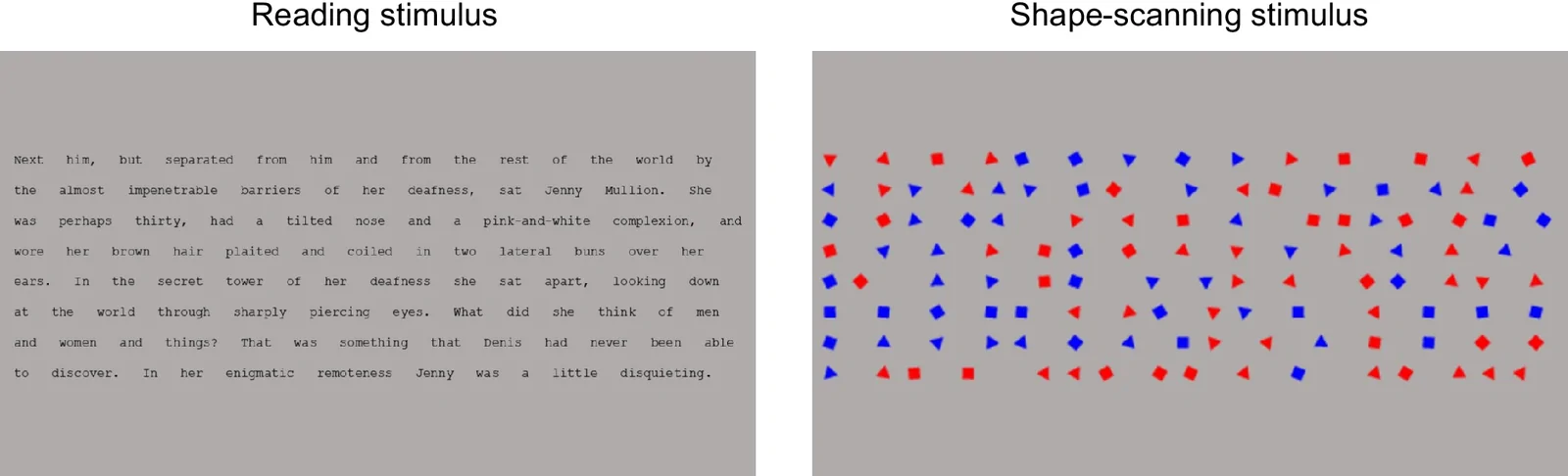
Examples of reading and shape-scanning stimuli. The text sample for the reading stimulus is taken from *The Benefactress* (von Arnim, 1901). The shape-scanning stimulus is created to mimic the graphemic information of the text, which is done by placing a shape at the corresponding position of every fourth character in the text. Colours and shapes mimic the parafoveally and foveally processed textual information, respectively. (Colour figure online)

We propose and employ an adjusted version of the Landolt paradigm to investigate differences in eye movement behaviour between reading and shape-scanning. In the Landolt paradigm, participants have to fixate on most of the rings, as it is difficult to assess the relevance of a ring (i.e., whether it has an opening or not) from parafoveal vision. This presents a considerable difference to reading, where parafoveal information allows participants to skip even whole words, leading, for example, to only 35% of function words being fixated on (Rayner, 1998). To better mimic this property of reading stimuli, we adapt the Landolt paradigm by letting participants scan arrays of geometric shapes, the layout of which spatially matched with a reading stimulus (Fig. 1), and count the number of a certain shape–colour combination. The shapes in the shape-scanning stimuli are either triangles or squares, each coloured either red or blue. The different colours and shapes mimic characteristics of natural language: different colours allow for making distinctions in the peripheral view, similar to perceiving graphemic information during reading (Henderson et al., 1987, 1989; Rayner, 1975a, b, 1998, 2009a), whereas different forms necessitate fixating on a shape to determine whether it is a target, because is necessary when reading longer words in a text (Fiebelkorn & Kastner, 2019; Rayner, 2009a). Thus, for the shape-scanning task, we achieve that some points that appear potentially informative in the periphery must first be fixated on to evaluate their actual relevance. The shape, colour, and rotation of each object are randomised. We specifically select red and blue to cause as few problems as possible for participants suffering from colour blindness.

Otherwise, we follow the Landolt paradigm by giving the shapes a size comparable with that of the characters in the text and arranging them in lines, much like words in a text. The characters of the text serve as a reference for the positions of the shapes in the shape-scanning stimuli that mimic the graphemic information of the text. A shape is placed at the position of every fourth character. Since spaces reduce graphemic density, we do not include them when determining the positions of geometric shapes. All other characters (i.e., dots, commas, and colons) are considered when determining the positions of the shapes.

We conducted a preexperiment to determine the optimal spacing for the geometric shapes in the shape-scanning stimuli (results publicly available in the study’s repository). Three female volunteers participated in the preexperiment, all of whom were students at the Technical University of Darmstadt. In the preexperiment, we presented the reading stimuli along with shape-scanning stimuli in three configurations: shapes at every third character, every fourth character, and every fifth character. After each stimulus, we ask a brief question to verify whether participants performed the task with sufficient attention. To this end, a multiple-choice question with four answer options asks about a piece of information from the text or the number of red triangles from the shape-scanning stimulus. We only consider those trials in which participants answered correctly when evaluating the preexperiment. The participants needed an average of 43 s to read the texts. For the shape-scanning stimulus configurations, the time required to count all red triangles increased with the presence of more shapes. Participants needed 39 s in the fifth-character design, 42 s in the fourth-character design, and 47 s in the third-character design. Because the time for completing the shape-scanning stimuli in the fourth-character design is closest to the reading time of the texts, we place a geometric shape at the position of every fourth character. Creating 26 shape-scanning stimuli for the 26 reading stimuli yields a total of 52 stimuli for the experiment.

### Participants

We performed an a priori power analysis to determine our target sample size. For the expected effect size, we followed recent similar research that investigated eye movements during reading with a Landolt paradigm (Xia et al., 2024). Xia et al. (2024) base their power calculation on the effect size *d* = 0.87 as it was the lowest effect size achieved in a previous study (Liu et al., 2019). Under the standard criterion *α* = 0.05, this effect size yielded a minimum sample size of 14 participants to achieve statistical power of 80% for our study’s two-sided dependent-samples tests. A total of 27 native German speakers participated in the experiment, of whom 16 identified as female, 11 as male, and none as diverse. The data of no participant had to be excluded from the analyses. All participants were bachelor's students in psychology or cognitive science at the Technical University of Darmstadt. Their course requires them to participate in experiments for 30 hours. For their participation in this experiment, they were compensated with 1 hour for this requirement. All participants provided informed consent for participation. The experiment was approved by the Ethics Committee of the Technical University of Darmstadt (Application No. EK 81/2022). All experiments were performed in accordance with relevant guidelines and regulations.

We recorded the age of participants in a clustered manner to ensure their complete anonymity. Of the participants, 23 are between 18 and 23 years old, and four are between 24 and 29. The participants provide a self-assessment of their English proficiency. They rate it on a scale from 0 to 10, where 0 means they speak no English and 10 means they speak it at the level of native speakers. The average self-assessed English proficiency of the participants is 7.6. Regarding the native languages, no participants were native English speakers, and two reported having a second native language in addition to German.

### Equipment

The experiment is conducted with an EyeLink 1000 Plus eye tracker from SR Research Limited. We use the Experiment Builder software provided by SR Research for stimulus presentation, timing, and data recording. Identification of saccades and fixations is performed by the online parser integrated into the eye-tracking device. The parser employs thresholds for motion, velocity, and acceleration to identify saccades and fixations. We use the defaults recommended by the manufacturer for running cognitive experiments, with 0.15° for motion, 30°/s for velocity, and 9,500°/s^2^ for acceleration. Eye movements are recorded at 1000 Hz. We use a ThinkVision monitor from Lenovo, measuring 60 cm in width, 34 cm in height, and featuring a resolution of 1,280 × 720 pixels. The participant’s head is positioned 70 cm from the monitor, with a chin rest maintaining a constant distance from the screen and ensuring the same distance for all participants. The experiment is conducted in a soundproof cabin darkened from daylight with constant artificial lighting. The analyses are performed with Python’s SciPy and *statsmodels* packages. The plots are created with matplotlib in Python.

### Experimental procedure

Before participants see the first stimulus, we introduce them to the tasks and the experiment’s operation. An instruction text tells the participants to read the text stimuli carefully and that they will have to answer a question on each text. For the shape-scanning stimuli, participants are told to count the red triangles. As in experiments involving the Landolt paradigm (Corbic et al., 2007; Günther et al., 2012; Hillen et al., 2013; Zschornak et al., 2008, 2012) and other pseudoreading or mindless-reading experiments (Rayner & Fischer, 1996; Vitu et al., 1995), we explicitly instruct participants to look at the shapes from top left to bottom right, like lines of text. In a few cases where participants do not adhere to this procedure from the beginning, we instruct them again. At the latest, from the second shape stimulus, all participants scan the shapes like a text.

In each trial, the participant first sees a fixation target in the centre of the screen for the drift check. After the experimenter confirms the drift check, the participant views the stimulus. We randomise the stimuli to ensure that the same type of stimulus, reading or shape-scanning, is presented a maximum of three times consecutively. The order within a stimulus category is fully randomised. When the participant has read the text or counted the red triangles, they press the space bar. We decided to use this self-paced design to avoid putting any pressure on the participant and thus ensure natural reading behaviour. After the stimulus, the participants see a multiple-choice question about the content of the text (or about the number of red triangles in the shape-scanning stimuli) and four response options. Participants receive feedback on whether the answer was correct after entering it with keys 1 to 4.

### Data analysis

**Fig. 2:**
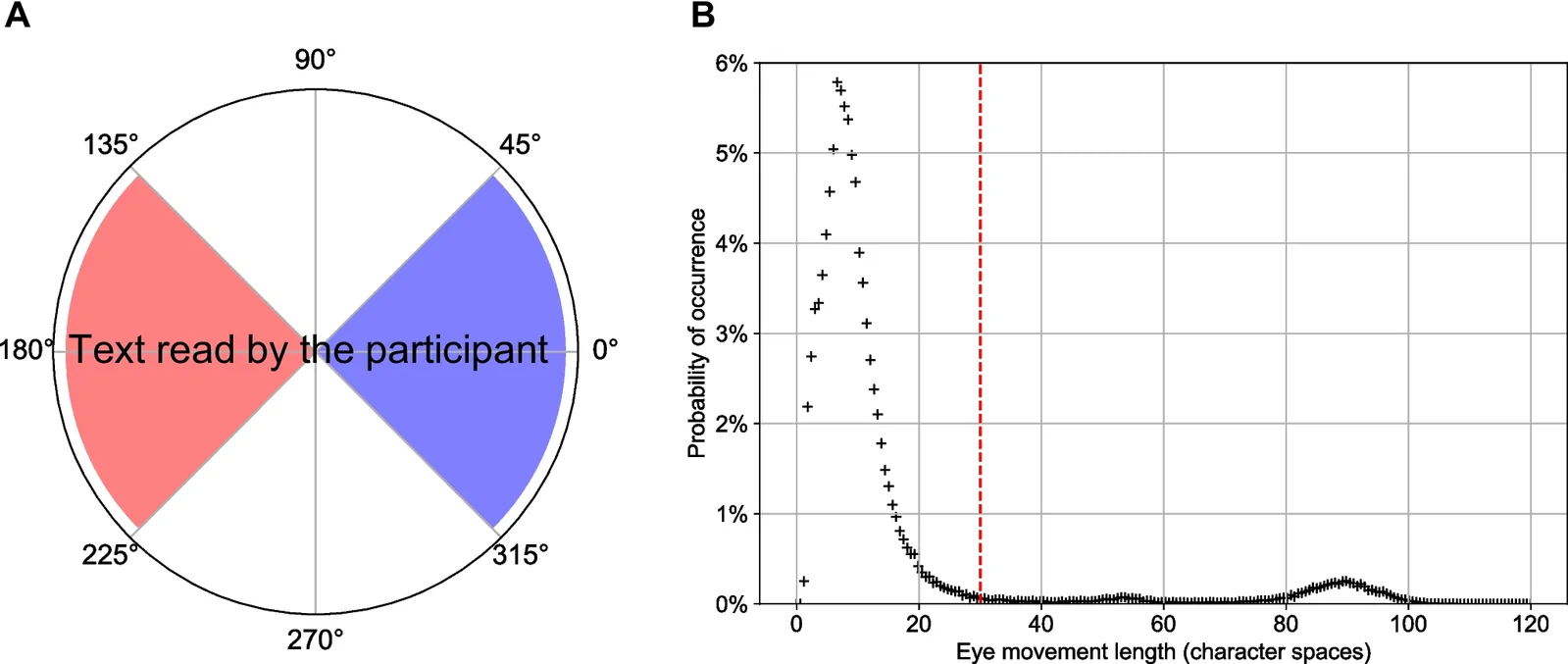
Inclusion criteria for eye movements in the analysis depending on angle and length. **A** Classification of eye movements into saccades and regressions depending on their direction. Saccades (blue) have a direction between 315° and 45°; regressions (red) have a direction between 135° and 225°. Eye movements in other directions are excluded. **B** Probability distribution for absolute eye-movement lengths. The *x-*axis shows the length of an eye movement measured in character spaces, and the *y*-axis shows the frequency with which an eye movement occurs. The red dashed line shows the threshold of 30 character spaces applied to remove return sweeps from the analyses. (Colour figure online)

First, we need to define the classification of eye movements. Considering a horizontal movement to the right as 0° with angles increasing clockwise (Fig. 2A), we define an eye movement as a saccade if its direction is between 315° and 45° (Fig. 2A, blue). The direction of a saccade is calculated using the positions of the initial fixation and the target fixation. We define the regressions like the saccades, except that their direction must be to the left. Regarding the angular representation, the direction of an eye movement must be between 135° and 225° for it to be considered a regression (Fig. 2A, red).

We exclude all other upward and downward eye movements, i.e., with directions between 45° and 135° or 225° and 315°, from the analyses. We know that upward eye movements while reading multiline text stimuli can serve, just like regressions, for reconsidering already-seen information. For our analyses, however, we restrict ourselves to horizontal eye movements and do not consider upward eye movements. The omission of these eye movements should not compromise our analyses, as they account for only 1.6% of our data. Consideration of these upward line jumps would certainly be worthwhile in future research. Furthermore, according to our definition of saccades and regressions, eye movements to the beginning of a new line would also be considered regressions. However, these *return sweeps* differ fundamentally from regressions since they do not involve refixating already viewed content. We can easily exclude return sweeps from regressions as we can identify them by their length. Since most return sweeps are about 90 character spaces long (Fig. 2B) due to the line length in our experiment, we can easily distinguish them from regular regressions, which are at most 30 character spaces long (Rayner, 1998).

Our analyses are only directed to eye movements smaller than 30 character spaces (Fig. 2B), or 250 pixels. Concerning the reading stimuli, we follow Rayner’s (1998) specification of 25 character spaces. The shape-scanning stimuli are based on the reading stimuli, but we cannot assume that the maximum length of an eye movement is also 25 character spaces. For this reason and better comparability of conditions, we set the limit for both types of stimuli to 30 character spaces (Fig. 2B, dashed line). Although we may still lose some data for the shape-scanning condition by this choice of threshold, an upper bound on the length of eye movements is important to us for two reasons. First, eye movements above this size occur less and less frequently, which means that our data have a high variance and are thus less robust. Second, larger regressions, for example, are caused by participants checking whether another line follows at the end of the last line. This behaviour would result in observing return sweeps in the data with lengths of less than 90 characters, which we do not want to examine. Thus, with a maximum of 30 characters per saccade or regression, we make a trade-off in which the data quality for reading stimuli is not diminished, and sufficient data for shape-scanning stimuli is retained. After removing eye movements longer than 30 characters, 77.7% of eye movements remain in the data.

In addition to the standard measures, we investigate relational dependencies between eye movements. We employ the previously established relative saccade length (Fabian, 2025; i.e., the length of a saccade measured as a factor of the preceding eye movement’s length). First, we take the temporally recorded and sorted fixation positions to calculate the absolute eye movement distances as the Euclidean distance between two successive fixations. We determine the relative lengths by dividing each length of an eye movement by the length of the preceding eye movement. Thus, a trial containing *n* fixations results in *n* − 1 absolute saccade lengths and *n* − 2 relative saccade lengths. For all participants and trials, the 111,350 recorded saccades yield 107,558 relative saccade lengths. In this analysis, we separate saccades and regressions to refine Fabian's (2025) results. The relative length of a saccade is given as a multiple of the preceding saccade, a regression’s length in relation to the preceding regression. Expressing the lengths of eye movements relative to each other provides a unit-independent measure of eye-movement lengths that is not affected by transformations of the absolute lengths, e.g., from degrees of visual angle to character spaces.

To determine the most probable relative lengths, we apply a Gaussian filter to the distributions of relative lengths. For individual data points of the distributions of relative lengths, there are sometimes only a few measurements available, especially if we want to determine the most probable relative length for individual participants. Smoothing the distribution allows the recognition of underlying trends. The most probable relative length is the global maximum of this smoothed distribution. We calculate the local maxima using the local minima of the derivative of the distribution. The local maximum with the highest value is the global maximum. We only smooth the probability distributions for estimating the maxima. We use the unsmoothed probability distribution for all other analyses and the visualisations.

The alpha level is 0.05 for all statistical tests. Reported *p* values are corrected with the false discovery rate according to Benjamini–Hochberg (Benjamini & Hochberg, 1995). For *t* tests and Wilcoxon signed-rank tests, Cohen’s *d*_z_ (Cohen, 1988; Lakens, 2013) is calculated to assess the effect size. For the Friedman test, Kendall’s *W* (Kendall & Smith, 1939) is applied to assess the effect size. As we do not consider between-subject factors, all statistical tests consider the groups as within-subject factors.

## Results

In the following, we apply the terminology used in reading research (Engbert et al., 2005; Rayner, 1998, 2009a, b). In reading experiments, eye movements in the direction of reading, in English from left to right, are called saccades. Eye movements directed opposite to the reading direction are called regressions. We use the terms *saccade* and *regression* because all stimuli used in the experiment are intended to be viewed like texts. In studies investigating general gaze behaviour, the terms pro-saccade, anti-saccade, or anti-parallel saccade are more common to distinguish various eye movements (Hallett, 1978; Kümmerer et al., 2022; Munoz & Everling, 2004). Since reading is a main part of the experiment and we want to use consistent terminology, we use the terms saccade and regression.

### Standard measures

The fixation durations are shorter during reading (mean = 219.28 ms, *SD* = 121.83 ms) than during shape-scanning (mean = 232.22 ms, *SD* = 131.19 ms). As the Shapiro–Wilk tests for the fixation durations during reading (*p* = 0.660) and shape-scanning (*p* = 0.939) indicate normally distributed data and Levene’s test indicates variance homogeneity (*p* = 0.239), we apply a two-tailed *t* test. The two-tailed *t* test shows that the difference in fixation durations during reading and shape-scanning is significant (*t* = −2.949, *p* = 0.007, *d*_z_ = −0.567).

For the absolute saccade lengths measured in degrees of visual angle, we differentiate between saccades and regressions. We report the lengths in degrees of visual angle since the presentation screen’s resolution can influence lengths reported in numbers of pixels. For our experimental setup, one degree of visual angle spans approximately 45 pixels. Saccades are smaller during reading (mean = 3.09°, *SD* = 2.12°) than during shape-scanning (mean = 4.08°, *SD* = 2.64°). To test for significance, we apply the Wilcoxon signed-rank test, as the Shapiro–Wilk test does not indicate a normal distribution for the saccades during reading (*p* = 0.012). The Wilcoxon signed-rank test reveals that the difference in saccade lengths during reading and shape-scanning is significant (*W* = 1, *p* < 0.001, *d*_z_ = −2.162). Similar to the saccades, regressions are smaller during reading (mean = 7.73°, *SD* = 10.15°) than during shape-scanning (mean = 9.75°, *SD* = 9.66°). The Shapiro–Wilk test for the regression lengths during reading does not indicate a normal distribution (*p* = 0.004). The difference in regression lengths between reading and shape-scanning is significant, as indicated by a Wilcoxon signed-rank test (*W* = 44, *p* < 0.001, *d*_z_ = −0.812).

### Relative eye-movement lengths

**Fig. 3:**
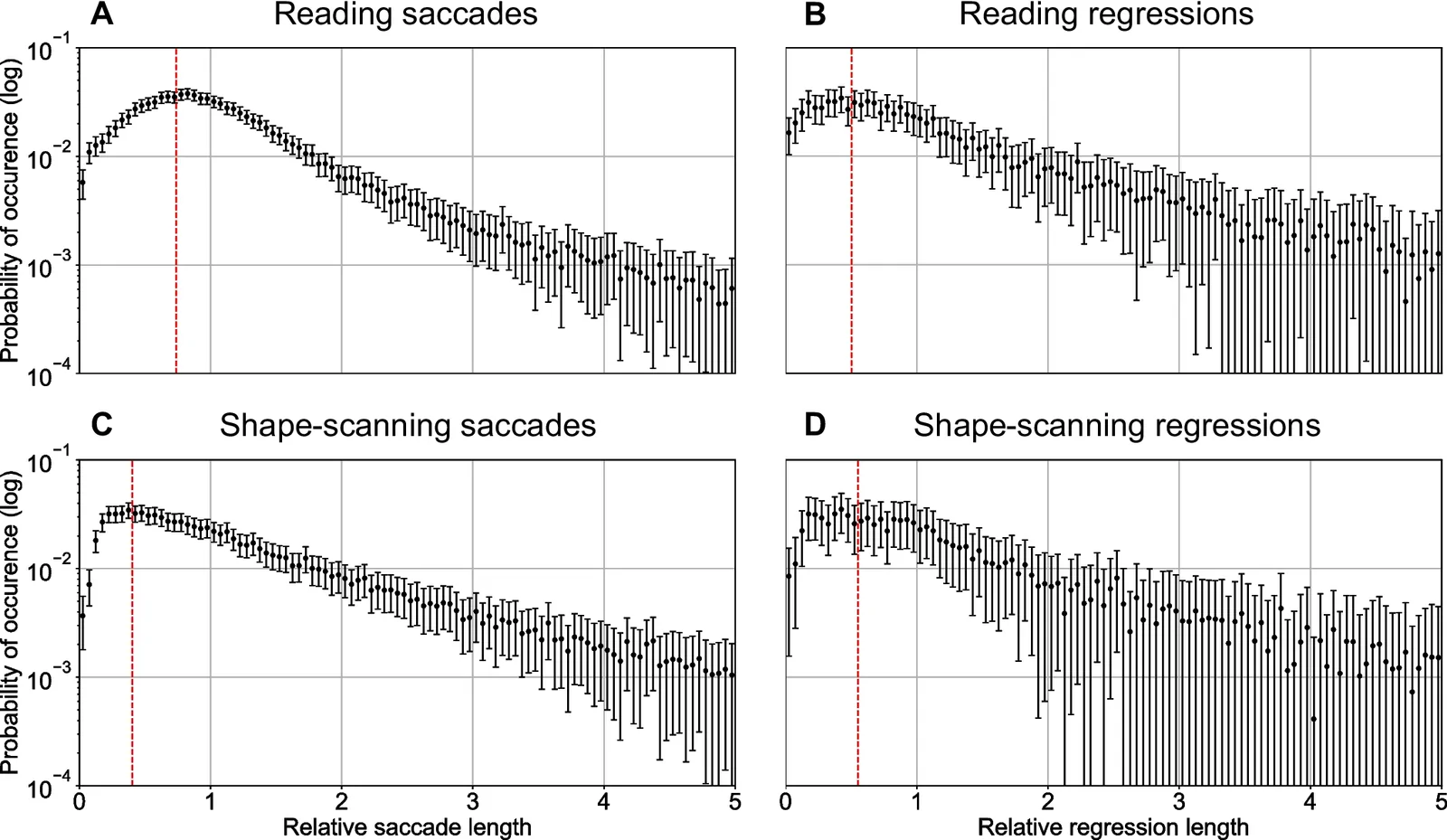
Distributions of relative eye-movement lengths during reading and shape-scanning. The eye movements are divided into saccades during reading (**A**), regressions during reading (**B**), saccades during shape-scanning (**C**), and regressions during shape-scanning (**D**). The *x*-axis shows the length of an eye movement as a multiple of the preceding eye movement’s length, and the *y*-axis shows, on a logarithmic scale, the frequency with which an eye movement occurs. The red dashed lines show the maxima for the functions approximated from the distributions. For reading, the most probable relative lengths are 0.738 for saccades and 0.503 for regressions, and for shape-scanning, 0.405 and 0.551, respectively. Error bars indicate the standard deviation. (Colour figure online)

Following Fabian (2025), we use relative eye-movement lengths to describe participants' gaze behaviour. Figure 3 shows the probability of a relative length as a function of its size for the saccades and regressions in reading and shape-scanning. For the relative lengths, we express the length as a factor obtained by dividing a saccade’s length by the preceding saccade's length. Note that we deliberately deviate from Fabian's (2025) illustration, in which both axes are scaled logarithmically to illustrate the power law relationship. In our case, only the y-axis is logarithmically scaled. We scale the *x*-axis linearly so that the differences in the maxima of the distributions are visible.

The distributions of the relative lengths of eye movements for reading and shape-scanning show similarities. The maximum (i.e., the peak probability of the individual factors) is about the same in all distributions at approximately 3.5%. More important than the peak probability itself is the position of the peak probability (Fig. 3, red dashed line), as it shows the consistency of the eye-movement lengths. We find that the average relative length of an eye movement is close to the value 1 for saccades during reading (1.001), saccades during shape-scanning (0.998), regressions during reading (0.997), and regressions during shape-scanning (1.014). The peaks’ positions of the relative saccade lengths show a clear difference between the tasks. For saccades during reading (Fig. 3A), the mean peak position is 0.738 (*SD* = 0.122). During shape-scanning, the most probable relative saccade length is considerably smaller (Fig. 3C) with a mean of 0.405 (*SD* = 0.082). Contrary to the saccades, the peak positions of relative regressions are similar for reading and shape-scanning. Here, the mean peak positions across all participants are 0.503 (*SD* = 0.197) for reading (Fig. 3B) and 0.551 (*SD* = 0.223) for shape-scanning (Fig. 3D). When reading, the average peak position of the relative saccades is greater than that of the relative regressions. In shape-scanning, the average peak position of the relative saccades is smaller than that of the relative regressions.

We conduct statistical tests for the peak positions of the participants’ distributions to investigate the significance of these results. We cannot assume normally distributed data, as Shapiro–Wilk tests for the saccades in reading (*p* = 0.006) and the regressions in shape-scanning (*p* = 0.018) are significant. Variance homogeneity is also not given, as indicated by Levene's test (*p* = 0.002). Given these test results, we apply the nonparametric Friedman test to our repeated-measures data. The Friedman test shows significant differences between the peak positions of the four groups, χ^2^(3) = 31.17, *p* < 0.001, Kendall’s *W* = 0.096.

We perform Wilcoxon signed-rank tests to examine the significant differences more thoroughly. The test results show that the peak positions for relative saccades and regressions differ significantly for reading (*W* = 33.5, *p* < 0.001, *d*_z_ = 1.027) and shape-scanning (*W* = 60, *p* = 0.006, *d*_z_ = −0.659). When comparing the task types, we find that the average peak position of relative saccades is significantly larger during reading than shape-scanning (*W* = 0, *p* < 0.001, *d*_z_ = 2.607). We observe no significant difference in the relative regression lengths of both tasks (*W* = 141.5, *p* = 0.572, *d*_z_ = −0.133).

## Discussion

For this work, we conducted an experiment to investigate the vision–language interface by comparing the visual behaviour during reading and shape-scanning. In the experiment, participants read texts in one half of the trials and counted all red triangles in collections of shapes in the other half. The standard measures of average absolute saccade lengths and fixation durations show that the saccades, regressions, and fixation durations are significantly longer during shape-scanning than during reading. These results are partly in contrast to findings from other experiments on scanning nonreadable strings. Similar to our results, Rayner and Fischer (1996) found that scanning involves longer fixation durations, 324 ms for scanning and 275 ms for reading. Fixations in our experiment are comparable though shorter, with 232 ms for scanning and 219 ms for reading, which are both near the average ranges of 180–275 ms and 225–250 ms, respectively (Rayner, 2009a). However, Rayner and Fischer (1996) also report comparable average saccade lengths of around seven character spaces or 1.75° of visual angle for reading and scanning and, analysing the length distributions in detail, argue that scanning leads to more short saccades. In contrast, in our experiment reading and scanning lead to saccades of 3.09 and 4.08 degrees of visual angle, respectively, which are also longer than the respective averages of 2° and 3° (Rayner, 2009a).

We suspect that the different findings for the saccade lengths stem from that fact that using absolute saccade lengths as a measure depends on the exact stimulus configuration. An important stimulus-driven mechanism that influences absolute saccade lengths is visual crowding (Bouma, 1970) and the closely related concept of the visual span (Pelli et al., 2007). Crowding refers to the fact that objects in peripheral view are harder to identify if other elements are close to it. When crowding is strong due to small spacing and many items, the visual span shrinks (i.e., the number of items that can be recognized in a single fixation decreases), which requires the viewer to perform shorter saccades. Therefore, average saccade length depends on the spacing of items and the complexity of each item. Thus, if we had created the shape-scanning stimuli by replacing every third character with a shape instead of every fourth, the crowding would have been stronger and the visual span shorter. As a consequence, the saccades during shape-scanning would have been shorter and might have matched the saccades during reading. Similarly, if we had let participants perform a simple feature search (red objects) instead of a conjunction search (red triangles), the complexity of relevant information would have been lower, thus presumably leading to longer saccades during shape-scanning. Thus, absolute saccade lengths appear too stimulus-dependent for general statements on how visual behaviour compares for reading and shape-scanning. Any statement about absolute saccade lengths depends on the experimental design and exact stimulus configuration.

Beyond comparing established eye movement metrics, we also consider relative eye-movement lengths (Fabian, 2025) for a less stimulus feature-dependent comparison of reading and shape-scanning. By dividing the length of a saccade by the previous saccade’s length, the length is expressed as a factor of the previous saccade’s length. The same is done separately for the regressions. This transformation of absolute lengths into relative lengths enables us to investigate the vision–language interface independently of the stimulus configurations. To achieve this, we calculate the probability distributions of the relative lengths of saccades and regressions for reading and the shape-scanning task. As the results of Fabian (2025) show, the distributions of relative eye-movement lengths possess a power-law tail across a wide variety of tasks and stimuli. However, the results also show slight deviations in the slope of that tail, with power-law coefficients ranging from 1.72 to 2.30 (Fabian, 2025). Thus, we hypothesised that this regularity, and in particular the possible deviations therein, might prove valuable as a new measure for human eye-movement behaviour. With the hypothesised convergence of average relative eye-movement lengths towards the value 1, the power-law coefficient of the distribution’s tail would be linked to the position of the distribution’s maximum. As maxima at larger relative lengths necessitate steeper slopes at the distribution’s tail to achieve an average relative length of 1, the maximum’s position and tail’s slope are directly linked. With the distinction of eye movements into saccades and regressions, we achieve a more fine-grained investigation of how different tasks might affect saccades and regressions, separately.

First, our results show that the hypothesised principle of convergence of relative lengths towards one seems to hold. Assuming that eye movements do not lengthen in the course of reading or shape-scanning, the average relative eye-movement length must converge towards one. We observe that the values of all four eye movement types we examined are close to one. The average relative length of a saccade during reading is 1.001, of a saccade during shape-scanning 0.998, of a regression during reading 0.997, and of a regression during shape-scanning 1.014. These results provide the basis for interpreting the other results. Since the average relative eye-movement length converges to 1, the position of the peak probability of the relative lengths is provided with an interpretative value. If the peak position is closer to 0 and there are, therefore, more small relative lengths, then there must also be more large relative lengths so that the mean value is still 1. Many small and large relative eye-movement lengths indicate that the absolute lengths of successive eye movements are not consistent, but that they vary in length. In contrast, if the peak position is close to 1, the relative eye-movement lengths are more densely distributed around 1, indicating more consistent absolute lengths in gaze behaviour.

Second, we find that the distributions of relative saccade lengths differ significantly for reading and shape-scanning with regard to the most likely relative length of an eye movement. The most probable relative length is 0.738 for saccades in reading and 0.405 for saccades in shape-scanning. In conjunction with the convergence of the average relative length towards 1, these results show that the lengths of saccades are more consistent in reading than in shape-scanning. Thus, gaze shift consistency in the target direction appears to differ between reading and shape-scanning, suggesting that the vision–language interface imposes constraints on saccadic planning. However, we do not observe this effect in the regressions, where the peak positions for reading and shape-scanning do not differ significantly, with values of 0.503 and 0.551, respectively. Hence, regressions during reading and shape-scanning do not appear to differ in terms of their length consistency. When participants are uncertain about the content seen in reading or shape-scanning, the behaviour of regressing seems to be independent of the task and stimulus. However, we must also note that several saccades are usually carried out between two regressions. Most regressions are, therefore, not performed in direct succession, and their contextual relationship is not necessarily guaranteed.

Our results for the relative saccade length distributions, which capture saccade-to-saccade variability, can be interpreted in line with neurocognitive theories and empirical findings on the vision–language interface. Although reading and non-word scanning underlie the same neural eye-movement network (Choi et al., 2014; Choi & Henderson, 2015; Hillen et al., 2013), we observe differences in the consistency of subsequent saccade lengths. The difference stems from the presence of linguistic information, as the lexical processing during reading imposes rapidly updated top-down constraints on saccade planning, a dependency that is also incorporated in established computational models of reading behaviour, like E-Z Reader (Pollatsek et al., 2006; Rayner, 1998; Rayner et al., 2004; Reichle et al., 1998, 2003) or SWIFT (Engbert et al., 2005). In contrast, when tasks do not require lexical processing, saccadic planning depends more on immediate decisions based on the stimulus’s visual salience, which leads to faster eye movements (Thakkar et al., 2015; van Zoest et al., 2004). Although the saccade planning is task-specific, our results for the relative regression lengths reveal similar behaviour for both tasks. Neuroanatomically, the medial frontal cortex has been shown to be involved in task-independent control of corrective saccades (Donahue et al., 2013; Godlove et al., 2011; Sajad et al., 2019). In addition to the medial frontal cortex, the anterior cingulate cortex is especially involved in conflict monitoring and cognitive control (Carter et al., 1998; Donahue et al., 2013; Kolling et al., 2016). The interaction between task-specific saccade planning and domain-general execution of corrective regressions could explain the observed differences and similarities between gaze behaviour during reading and shape scanning on a neural basis. Thus, the relative saccade length distributions might provide a novel way for revealing this connection between observable gaze behaviour and the underlying neural structures.

The results show that relative saccade lengths allow for assessing a component of human gaze behaviour that the absolute saccade lengths do not capture: the dynamics of gaze behaviour. The absolute measures indicate that saccades and regressions are significantly longer during shape-scanning than during reading. However, the relative saccade length distributions show that the dynamics of regressions are almost identical during both tasks. For the saccades, the different peak probabilities of relative saccade lengths indicate that saccade lengths during reading are more consistent than during shape-scanning. Distributions of relative saccade lengths allow for this additional comparison, as they capture the relations between saccade lengths by introducing sequential spatial dependencies. Simply considering the variability of absolute saccade lengths does not capture this dependency. For example, if a viewer performed many small saccades followed by many large saccades, the overall variability would be high. However, the relative saccade length distribution would reveal that the dynamics of this gaze behaviour are very consistent, as the saccade-to-saccade differences are small.

The main limitation of the current study is that its experiment design does not allow for a differentiation between the influence of task and stimulus. In the reading trials, participants read texts, and in the shape-scanning trials, they count shapes in arrays of geometric shapes. Thus, the task and stimulus both differ between the conditions. However, we consider this conflation of task and stimulus as inherent to the investigation of the vision–language interface, as removing linguistic information from a reading stimulus inevitably prevents genuine reading from being performed as the task. For reading stimuli, it seems impossible to change only the stimulus or the task. If only the task is changed and participants are, for example, asked to search for specific words in a text, they will still perform lexical and orthographic-phonological processing (i.e., parts of reading; Rayner, 1998; Schotter et al., 2012). Thus, keeping the text as a stimulus confounds any task with reading processes. Similarly, if one wants to change only the stimulus and keep reading as the task, one would have to introduce a new sign system for the stimuli. Only if viewers can ascribe meaning to strings of symbols, one can refer to the task as reading (de Saussure, 1966; Eco, 1979; Iser, 1980; Rosenblatt, 1978). However, a new sign system would correspond to a text in another language, which in turn would make the stimulus a text. Therefore, we consider it unavoidable that removing linguistic information from a text entails changing the task.

## Conclusions

In this work, we showed how relative eye-movement lengths provide novel insights into the vision–language interface by highlighting similarities and differences in human visual behaviour when reading a text and scanning a nonlinguistic display. First, we provided initial evidence for the axiom of relative eye-movement lengths, that the average relative length converges towards 1 throughout a task. Second, we demonstrated that reading influences gaze guidance in the target direction when compared with the scanning of a nonlinguistic display. Overall, saccades during reading appeared to be more consistent, and the absolute lengths of successive saccades were more similar than during shape-scanning. Thus, the content and task modified the fundamental mechanism of relative eye-movement lengths for gaze guidance in the target direction. By contrast, regression behaviour appeared to be unaffected by content and task. Our results are in line with neurocognitive findings on the vision–language interface. Beyond confirming previous research, we see our results as initial evidence for how distributions of relative eye-movement lengths can be used to provide a new view on human gaze behaviour. In contrast to absolute lengths, relative lengths offer insights into the dynamics of gaze behaviour. The convergence of average relative lengths towards 1 can be used to operationalise the distribution’s peak as a measure of gaze behaviour dynamics. Furthermore, although comparisons of absolute eye-movement lengths might be affected by transformations between degrees of visual angle and character spaces, relative eye-movement lengths do not depend on the units of the underlying absolute eye-movement lengths. Thus, applying this novel measure of gaze behaviour dynamics is not limited to investigations of the vision–language interface. For any visual task, the eye-movement behaviour can be described via its distribution of relative eye-movement lengths. This also allows for new comparisons between any two types of tasks that may not have seemed comparable before, potentially opening up a new avenue for research on human gaze behaviour.

## Data Availability

The materials used in the experiment and the datasets generated by the experiment and analysed during the current study are available on the Open Science Framework: 10.17605/OSF.IO/HNU32

## Code availability

The custom code used for conducting the analyses reported in the current study is available on the Open Science Framework: 10.17605/OSF.IO/HNU32
